# Bridging Gaps: Analyzing Breast Imaging-Reporting and Data System (BI-RADS) 0 Rates and Associated Risk Factors in Disproportionally Affected Communities

**DOI:** 10.7759/cureus.61495

**Published:** 2024-06-01

**Authors:** Mona P Roshan, Rebecca O'Connell, Maheen Nazarally, Pura Rodriguez de la Vega, Prasad Bhoite, Julia Bisschops, Marcia Varella

**Affiliations:** 1 Radiology, Florida International University, Herbert Wertheim College of Medicine, Miami, USA; 2 Internal Medicine, Florida International University, Herbert Wertheim College of Medicine, Miami, USA; 3 Medical and Population Health Sciences Research, Florida International University, Herbert Wertheim College of Medicine, Miami, USA; 4 Humanities, Health, and Society, Florida International University, Herbert Wertheim College of Medicine, Miami, USA; 5 Family Medicine, Florida International University, Herbert Wertheim College of Medicine, Miami, USA

**Keywords:** community health, preventive medicine, underserved, breast cancer, mammography

## Abstract

Introduction

Disparities in access to breast cancer screening led to the creation of the Linda Fenner 3D Mobile Mammography Center (LFMMC), successfully increasing screening for uninsured women in Miami-Dade. However, a higher-than-expected rate of inconclusive mammograms (Breast Imaging-Reporting and Data System (BI-RADS) 0) was found, which could lead to unnecessary procedures, stress, costs, and radiation.

Methods

In this retrospective cross-sectional study, we analyzed data from 3,044 uninsured women aged over 40 (younger if positive family history of breast cancer) from Miami-Dade without breast symptoms or breast cancer history. Women’s demographic characteristics, primary language spoken, body mass index (BMI), use of hormone replacement therapy and birth control, history of benign biopsy, breast surgery, family breast cancer, and menopausal status were assessed as potential risk factors for an inconclusive (BI-RADS 0) screening mammogram result. Multivariable logistic regression analyses were used to evaluate associations.

Results

The average age of women was 51 years (SD = 9); 59% were White, and 30% were African American. The overall frequency of BI-RADS 0 was 35%. Higher odds of BI-RADS 0 were found for women who were younger, single, premenopausal, and with benign biopsy history. Conversely, obesity and breast implant history decreased the odds of BI-RADS 0.

Conclusion

We found a high frequency of BI-RADS 0 in the LFMMC sample. Potential reasons include a higher risk for breast cancer or a younger sample of women screened. Future research should explore radiologists’ reasoning for assigning BI-RADS 0 results and testing alternative screening strategies for younger women.

## Introduction

Breast cancer is one of the leading causes of cancer death in women, with an estimated worldwide incidence of approximately 3.2 million new cases per year by 2050 [1,2]. Screening women for breast cancer via mammography has led to improved breast cancer survival rates. In the United States, the American College of Radiology recommends annual screening mammography beginning at age 40 for women at average risk [3].

Despite an increase in early detection of breast cancer across all groups, disparities remain regarding breast cancer screening and prognosis. Non-Hispanic white women are more likely to have access to breast cancer screening than Hispanic and African American racial groups [4,5]. The Linda Fenner 3D Mobile Mammography Center (LFMMC) was established to improve access and bridge this gap by providing free screening mammograms for uninsured women in Miami-Dade County [6]. Since 2014, the LFMMC has successfully performed over 6,500 free screening mammograms. 

The radiologist of the LFMMC uses the American College of Radiology’s Breast Imaging-Reporting and Data System (BI-RADS) to assess mammography in a standardized way [7]. Negative test results include BI-RADS 1, a normal finding, and BI-RADS 2, findings that are not cancerous, such as benign calcifications, masses, or lymph nodes in the breast [8]. Mammography results are sometimes considered to have a BI-RADS category of 0, meaning their mammography results are inconclusive and require further diagnostic testing [9]. Findings of BI-RADS 0 might lead to patient stress, increased costs, a low rate of patient satisfaction, and a low likelihood of return for routine breast cancer screening in the next year [10]. Therefore, minimizing the frequency of unnecessary inconclusive exams is critical. The accepted national performance benchmark percentage of BI-RADs 0 is approximately 5-12%[11]. Yet, the percentage of BI-RADs 0 on the LFMMC has been reportedly higher since inception. The reasons for such high rates of inconclusive results were unclear. LFMMC prioritizes quality assurance, minimizing technical recalls and ensuring 100% congruence in double reads for select cases, in line with accreditation requirements. This focus ensures reliable mammogram interpretations and underscores LFMMC's commitment to quality breast cancer screening.

In a study in Mexico between 2014 and 2017, the increase in BI-RADS 0 from 9.5% to 35.4% was attributed to differences in radiologist experience [12]. At the LFMMC, one radiologist has read the screening mammograms since its inception; thus, this aspect would not apply to our study. In Nigeria, a study of diagnostic mammography revealed an association between high breast density and inconclusive outcomes [13]. Yet, these findings contrast with a Taiwanese study that failed to find that breast density was associated with BI-RADS categorization [9]. Furthermore, a retrospective study in Delhi, India, emphasized that improper breast positioning, identified in 2.9% of mammograms, was linked to decreased sensitivity and increased inconclusive results [14]. Based on our current understanding, there is a lack of recent studies conducted in the United States at free-standing or hospital-based sites that examine risk factors for inconclusive mammograms. Consequently, we have drawn upon international examples to fill this gap in the literature. This approach not only justifies the utilization of global data but also emphasizes the importance of our research in elucidating contributing factors specific to the United States.

Prior studies assessing the risk factors for inconclusive mammography results were inconsistent and likely not externally valid for mobile centers in urban United States. The present study aimed to determine the frequency of BI-RADS 0 results in screening mammograms at a mobile program, the LFMMC, and explore potential risk factors for inconclusive mammograms in that setting. Our goal was to identify important clinical and demographic factors that may predispose individuals to BI-RADS 0 results using the LFMMC data.

## Materials and methods

**Figure 1:**
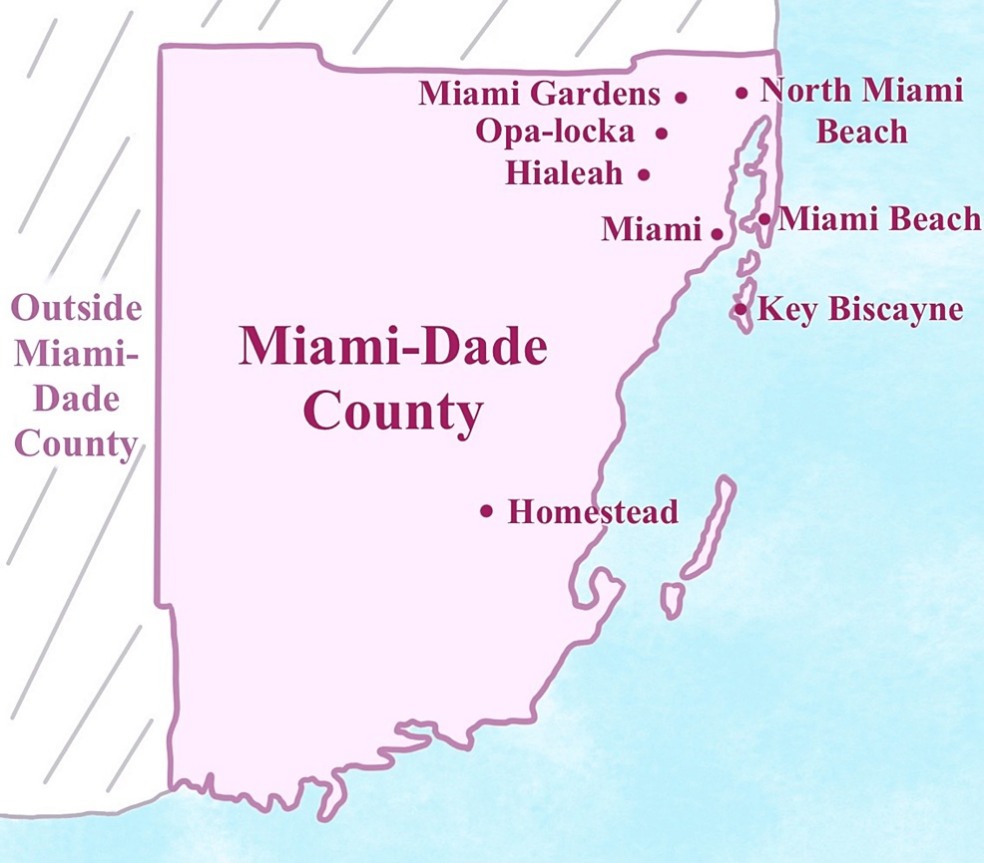
Sites visited by the Linda Fenner 3D Mobile Mammography Center. Illustrated by Sara Kian BA BS at UC San Diego

We performed analyses of cross-sectional data of over 5000 women participating in the LFMMC from 2014 (the year LFMMC opened) to 2022. Of those, 3,044 women had relevant data available (participant information was linked from three separate data sources: the LFMMC Appointment Report, Red Cap Report, and Mammo History Tech Note). The sample consisted of women who were 40 years or older (younger if positive for a family history of breast cancer), uninsured, and residents of Miami-Dade County. Women with a personal history of breast cancer, who were experiencing breast symptoms, or who were pregnant were excluded. Figure 1 illustrates the sites visited by the LFMMC.

Independent variables assessed included the participant’s age, race (White, African American, or other), ethnicity (Hispanic/Latino, or not Hispanic/Latino), marital status (married, single, widowed, or divorced), geographic area of residence, primary language spoken (English, Spanish, or other), BMI, history of current use of hormones and birth control pills, history of a previous benign biopsy result, surgical history of breast reduction or implantation, menopausal status (menopause or note based on last menstrual period), and family history of breast cancer. These variables were selected based on their potential impact on breast cancer risk and screening outcomes, encompassing demographic factors (age, race, ethnicity, marital status, geographic area, primary language), clinical history (BMI, hormone and birth control use, benign biopsy history, breast surgery history, menopausal status), and genetic predisposition (family history of breast cancer).

To provide context on the geographic areas of residence, the mean household income in dollars for Homestead is $57,739, Hialeah is $49,531, Opa Locka is $30,101, Miami Gardens is $56,071, Miami is $54,858, Miami Beach is $65,116, North Miami Beach is $56,071, and Key Biscayne is $173,015. These figures are based on the American Community Survey five-year data measured between 2018 and 2022 [15]. The dependent variable was a screening mammogram radiology report of the BI-RADS as inconclusive (BI-RADS 0), as opposed to normal screening results (BI-RADS 1 & 2).

Statistical analysis

The frequency of BI-RADS was assessed and compared according to each risk factor. Statistical significance for the proportions in each group was assessed using chi-squared tests. Associations were evaluated by uni- and multivariable logistic regression analyses. We estimated odds ratios and reported the corresponding 95% confidence interval. The p-value was considered significant for a p-value of 0.05 for a two-tailed hypothesis testing. Stata v 17 software was used for all analyses. The FIU Health Sciences Institutional Review Board approved this study (# IRB-17-0142-AM05 and Reference # 105438).

## Results

From 2014 to 2022, the LFMMC conducted mammography screenings on 5,017 women. Women between the ages of 35 and 88 years were included if the information was available in the datasets: the LFMMC Appointment Report, Red Cap Report, and Mammo History Tech Note. Women below the age of 40 were included if positive for a family history of breast cancer. In contrast, women above 75 were included to ensure a comprehensive analysis across various age groups. This decision aligns with the American Cancer Society guidelines, which recommend continuing screening for women aged 55 and older who are in good health and expected to live at least 10 more years.

**Figure 2:**
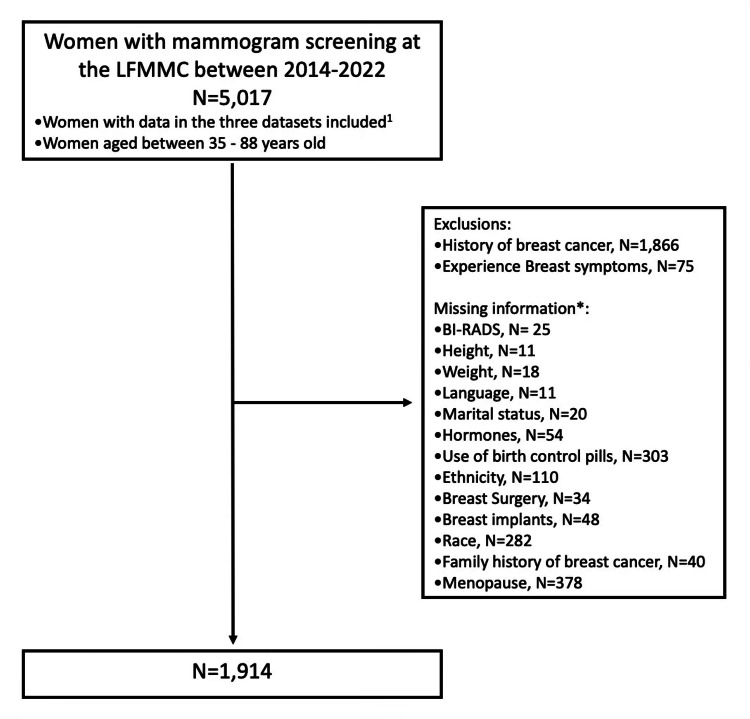
Flow diagram showing the selection of participants included in this study LFMMC: Linda Fenner 3D Mobile Mammography Center; BI-RADS: Breast Imaging-Reporting and Data System 1: Mammovan Appointment Report, Red Cap Report, and Mammo History Tech Note. *: Missing information extends to more than one variable for some women.

Exclusion criteria included women with a history of breast cancer (n = 1,866) and breast symptoms (n = 75), which produced 3,044 eligible women. These women were excluded to prevent confounding factors that could bias the study results and because these patients do not typically receive screening mammograms, aligning with the American College of Radiology's recommendation for annual mammographic screening starting at age 40 for women at average risk for breast cancer. This approach ensures a more accurate analysis of the clinical and demographic factors associated with BI-RADS 0 scores. Due to missing information within the various independent variables because of changes over time since the conception of the LFMMC, we also had to exclude 1,130 women from the analyzed women category. As a result, 1,914 LFMMC patients were analyzed (Figure 2).

Table 1 shows the baseline characteristics of eligible (n = 3,044) and analyzed (n = 1,914) adult female patients of the LFMMC. In the analyzed women, the average age was 51.5 years (SD = 9.1), with the majority being younger than 50 (53.3%; n = 1,020), White race (66.6%; n = 1,274), and Hispanic or Latino for ethnicity (67.7%; n = 1,296); most were married (39.2%; n = 748) or single (39.6%; n = 755). Most (73.4%; n = 1,405) lived in Miami and spoke Spanish as their primary language (59.5%; n = 1,139). The average BMI of the study participants is 29.6 (SD = 7.2), with the majority in the obese (43.2%; n = 826) or overweight (33.3%; n = 638) categories. Use hormone replacement therapy was reported by 3.1% (n = 60), while 2.8% (n = 53) use birth control pills, 6.0% (n = 114) had a previous benign biopsy, 9.1% (n = 175) had breast surgery, and 6.2% (n = 119) had breast implants. Family history of breast cancer was reported in 17%, and 53.0% (n = 1,015) were premenopausal. A total of 65% (n = 1,244) and 35% (n = 670) of the women had BI-RADS 1 & 2 and BI-RADS 0 screening mammography results, respectively. 

**Table 1:** Characteristics of eligible and analyzed females in the Linda Fenner 3D Mobile Mammography Center BI-RADS: Breast Imaging-Reporting and Data System; BMI: body mass index 1: Geographic areas were defined based on www.zip-codes.com. 2: BI-RADS classification of 0, 1, and 2 means inconclusive, negative, and benign, respectively. *Results reported as n and %, unless otherwise specified. **Seven (7) participants had marital status marked as “other” and were considered missing information on marital status.

Characteristics		All eligible n (%)* total n = 3,044		All analyzed n (%)* total n = 1,914	
Age (years), mean ± SD		51.1 ± 9.0	-	51.5 ± 9.1	-
Age in years, categories	38-49	1,664	54.7	1,020	53.3
	50-59	761	25.0	471	24.6
	60 and older	619	20.3	423	22.1
Race	White	1,791	58.8	1,274	66.6
	African American	924	30.4	610	31.9
	Other	47	1.5	30	1.6
	Missing	282	9.3		
Ethnicity	Hispanic or Latino	2,045	67.2	1,296	67.7
	Not Hispanic or Latino	889	29.2	618	32.3
	Missing	110	3.6	-	-
Marital status	Married	1,183	38.9	748	39.2
	Single	1,222	40.1	755	39.6
	Divorced, separated	456	15.0	305	15.9
	Widowed	156	5.1	106	5.6
	Missing	27	0.9	-	-
Geographic areas^1^	Homestead	188	6.2	126	6.6
	Hialeah	357	11.7	211	11.0
	Opa Locka	93	3.1	46	2.4
	Miami Gardens	46	1.5	35	1.8
	Miami	2,201	72.3	1,405	73.4
	Miami Beach	86	2.8	56	2.9
	North Miami Beach	55	1.8	28	1.5
	Key Biscayne	3	0.1	1	0.1
	Outside Miami-Dade	6	0.2	6	0.3
	Missing	9	0.3	-	-
Primary Language Spoken	English	832	27.3	511	26.7
	Spanish	1,794	58.9	1,139	59.5	
	Other	407	13.4	264	13.8	
	Missing	11	0.4	-	-	
BMI, mean ± SD		29.3 ± 7.5	-	29.6 ± 7.2	-
BMI, categories	Underweight	192	6.4	90	4.7
	Normal	561	18.6	360	18.8
	Overweight	989	32.8	638	33.3
	Obese	1,273	42.2	826	43.2
	Missing	29	0.95	-	-
Hormone use	Yes	92	3.0	60	3.1
	No	2,898	95.2	1,854	96.9
	Missing	54	1.8	-	-
Birth control pill use	Yes	76	2.5	53	2.8
	No	2,665	87.6	1,861	97.2
	Missing	303	10.0	-	-
History of prior benign biopsy	Yes	195	6.4	114	6.0
	No	2,849	93.6	1,800	94.0
History of breast surgery	Yes	298	9.8	175	9.1
	No	2,712	89.1	1,739	90.9
	Missing	34	1.1	-	-
History of breast implants	Yes	183	6.0	119	6.2
	No	2,813	92.4	1,795	93.8
	Missing	48	1.6	-	-
Family history of breast cancer	Yes	508	16.7	338	17.7
	No	2,496	82.0	1,576	82.3
	Missing	40	1.3	-	-
Menopausal status	Premenopause	1,415	46.5	1,015	53.0
	Postmenopause	1,254	41.2	899	47.0
	Missing	375	12.3	-	-
BI-RADS^2^	1 & 2	2,037	66.9	1,244	65.0
	0	982	32.3	670	35.0

Comparing the group of women who were eligible to those who were analyzed, the proportion of women in the 38-49 age group was slightly lower in the all-analyzed group compared to the eligible group, and the frequency of White and not Hispanic/Latino and married women were marginally higher in the analyzed group. Lastly, women who denied taking birth control pills, who had a family history of breast cancer, and who reported menopause were slightly higher in the sample analyzed.

Overall, 35% of the sample had a mammogram exam result of BI-RADS 0. The frequency of women classified as BI-RADS 0 (inconclusive) is highest in younger women (women between the ages of 38 and 49, 39.3%; p < 0.001), single women (38.8%; p < 0.05), women with a benign biopsy (43.9%; p < 0.05), women without breast implants (35.8%; p < 0.005), and premenopausal women (40.8%; p < 0.001) (Table 2). No statistically significant difference was found regarding the frequency of BI-RADS 0 classification for the following variables: race, ethnicity, language, BMI categories, hormone use, birth control pill use, breast surgery, and family history of breast cancer. Although BMI was found to be an insignificant variable when analyzed as a categorical variable, it was found to be significant as a continuous variable. Patients with inconclusive screening mammograms had an average BMI of 29.9 (SD = 7.4), statistically significantly higher than those with normal mammograms with an average BMI of 29.0 (SD = 6.8). 

**Table 2:** Characteristics of female patients of the Linda Fenner 3D Mobile Mammography Center and BI-RADS classification in a screening mammogram (N = 1,914) BI-RADS: Breast Imaging-Reporting and Data System; BMI: body mass index; Hx: history Results are shown in n and % unless otherwise specified. 1: BI-RADS classification of 0, 1, and 2 means inconclusive, negative, and benign, respectively.

Characteristics		BI-RADS^1^ N (%)*				
		Normal (1 & 2)		Inconclusive (0)		p-value
Age in years, mean ± SD		1,244	52.0 ± 9.1	670	50.4 ± 9.0	<0.001
Age in years, categories	38-49	619	60.7	401	39.3	<0.001
	50-59	337	71.6	134	28.5	
	60 and older	288	68.1	135	31.9	
Race	White	831	65.2	443	34.8	0.926
	African American	393	64.4	217	35.6	
	Other	20	66.7	10	33.3	
Ethnicity	Hispanic/Latino	856	66.1	440	34.0	0.161
	Not Hispanic or Latino	388	62.8	230	37.2	
Marital status	Married	502	67.1	246	32.9	0.014
	Single	462	61.2	293	38.8	
	Divorced/separated	201	65.9	104	34.1	
	Widowed	79	74.5	27	25.5	
Primary language spoken	English	330	64.6	181	35.4	0.964
	Spanish	741	65.1	398	34.9	
	Other	173	65.5	91	34.5	
BMI, mean ± SD		1,244	29.9±7.4	670	29.0±6.8	0.008
BMI, categories	Underweight	59	65.6	31	34.4	0.098
	Normal	226	62.8	134	37.2	
	Overweight	397	62.2	241	37.8	
	Obese	562	68.0	264	32.0	
Hormone use	Yes	35	58.3	25	41.7	0.272
	No	1209	65.2	645	34.8	
Birth control pill use	Yes	31	58.5	22	41.5	0.314
	No	1213	65.2	648	34.8	
History of prior benign biopsy	Yes	64	56.1	50	43.9	0.041
	No	1180	65.6	620	34.4	
History of breast surgery	Yes	105	60.0	70	40.0	0.146
	No	1139	65.5	600	34.5	
History of breast implants	Yes	92	77.3	27	22.7	0.004
	No	1152	64.2	643	35.8	
Family Hx of breast cancer	Yes	213	63.0	125	37.0	0.401
	No	1031	65.4	545	34.6	
Menopausal status	Premenopause	601	59.2	414	40.8	<0.001
	Postmenopause	643	71.5	256	28.5	

In Table 3, various factors were associated with a significantly increased likelihood of receiving a BI-RADS 0 result in the unadjusted model. Specifically, single women had 29% higher odds compared to married women (OR: 1.29; 95% CI: 1.05-1.60); women with a history of benign breast biopsy showed 49% higher odds compared to those without (OR: 1.49; 95% CI: 1.01-2.18); and premenopausal women had 73% higher odds compared to postmenopausal women (OR: 1.73; 95% CI: 1.43-2.10). Also, certain factors were significantly associated with a reduced chance of receiving a BI-RADS 0 result. Women aged 50-59 exhibited 39% lower odds (OR: 0.61; 95% CI: 0.48-0.78), and women aged 60+ had 28% lower odds (OR: 0.72; 95% CI: 0.57-0.92) compared to women aged 38 to 49. Additionally, women with breast implants demonstrated 47% lower odds of receiving a BI-RADS 0 result compared with those women without breast implants (OR: 0.53; 95% CI: 0.34-0.82). Moreover, the unadjusted model revealed a noteworthy association between BMI and the likelihood of receiving an inconclusive mammogram result. For each 1-unit increase in BMI, the odds of obtaining an inconclusive mammogram decreased by 2% (OR: 0.98; 95% CI: 0.97-1.00). We must note that we evaluated the functional form of BMI to ensure that modeling it as a (log)-linear variable is appropriate.

**Table 3:** Unadjusted and adjusted associations between selected characteristics and BI-RADS 0 screening result BI-RADS: Breast Imaging-Reporting and Data System; BMI: body mass index The model was adjusted for variables for which OR is shown.

Characteristics	Unadjusted	Adjusted-Model 1	Adjusted-Model 2
	OR (95% CI)	OR (95% CI)	OR (95% CI)
Ethnicity			
Hispanic or Latino	0.87 (0.71-1.06)	0.59 (0.39-0.88)	-
Not Hispanic or Latino	Reference	Reference	-
Marital status			
Married	Reference	Reference	Reference
Single	1.29 (1.05-1.60)	1.33 (1.07-1.65)	1.34 (1.08-1.66)
Divorced/separated	1.06 (0.80-1.40)	1.19 (0.89-1.60)	1.17 (0.88-1.56)
Widowed	0.70 (0.44-1.11)	0.80 (0.50-1.29)	0.82 (0.51-1.31)
Primary language spoken			
English	Reference	Reference	-
Spanish	0.98 (0.79-1.22)	1.44 (1.00-2.08)	-
Other	0.96 (0.70-1.31)	0.95 (0.67-1.33)	-
BMI (1 unit increase))	0.98 (0.97-1.00)	0.98 (0.96-0.99)	0.98 (0.96-0.99)
Hormone use			
Yes	1.34 (0.79-2.26)	1.26 (0.69-2.32)	1.25 (0.68-2.29)
No	Reference	Reference	Reference
Birth control pill use			
Yes	1.33 (0.76-2.31)	0.95 (0.50-1.80)	0.95 (0.50-1.80)
No	Reference	Reference	Reference
History of prior benign biopsy			
Yes	1.49 (1.01-2.18)	1.72 (1.16-2.56)	1.69 (1.14-2.50)
No	Reference	Reference	Reference
History of breast implants			
Yes	0.53 (0.34-0.82)	0.43 (0.27-0.67)	0.40 (0.26-0.63)
No	Reference	Reference	Reference
Family history of breast cancer			
Yes	1.11 (0.87-1.42)	1.19 (0.92-1.53)	-
No	Reference	Reference	-
Menopausal status			
Premenopause	1.73 (1.43-2.10)	1.90 (1.55-2.32)	1.85 (1.51-2.25)
Postmenopause	Reference	Reference	Reference

The variables for the logistic regression analyses were chosen based on those found significant in the unadjusted model analysis and those deemed significant by the authors. Collinearity among these variables was assessed, resulting in the exclusion of certain ones. For instance, age was excluded due to its collinearity with menopausal status. In the adjusted model 1 that included women’s ethnicity, marital status, primary language spoken, BMI, hormone usage, birth control pill usage, history of benign breast biopsy, presence of breast implants, family history of breast cancer, and menopausal status, being single compared to being married (OR: 1.33; 95% CI: 1.07-1.65), having a history of benign breast biopsy compared to not having one (OR: 1.72; 95% CI: 1.16-2.56), and being premenopausal compared to postmenopausal (OR: 1.90; 95% CI: 1.55-2.32) were associated with higher odds of receiving a BI-RADS 0 or inconclusive screening mammogram result. Moreover, being self-identified as Hispanic or Latino, as opposed to non-Hispanic or non-Latino, was associated with lower odds of obtaining a BI-RADS 0 result (OR: 0.59; 95% CI: 0.39-0.88). Similarly, for each incremental unit increase in BMI, there was an observed decrease in the odds of obtaining an inconclusive mammogram (OR: 0.98; 95% CI: 0.96-0.99). Lastly, women with breast implants displayed lower odds compared to those without implants (OR: 0.43; 95% CI: 0.27-0.67). In the adjusted model 2, we removed the variables ethnicity, language spoken, and family history of breast cancer, and the results remain similar to the adjusted model 1. 

## Discussion

The overall frequency of inconclusive screening mammograms with BI-RADS 0 classification in the LFMMC was 35%, considerably higher than the national average range of approximately 5-12% [11]. Significant factors associated with higher odds of inconclusive screening mammograms were identified, related to younger ages, such as premenopausal and single status. Lastly, the history of having a benign biopsy also had higher odds for a BI-RADS 0 mammogram result. 

A plausible explanation for higher odds of BI-RADS 0 screening results among younger patients is that younger, premenopausal women have higher breast tissue density than older women [16]. Higher breast tissue density has been associated with lower screening sensitivity [17]. A study in Nigeria [13] reported that high breast density was higher in subjects with inconclusive reports than those with low breast density. Additionally, a study in Taiwan found that the proportion of women with BI-RADS 0 classification was higher for women with premenopausal status, a history of breast surgery, and less than two abortions reported [9], aligning with our findings. 

Individuals with a self-reported history of benign breast biopsy had increased odds of receiving an inconclusive result from a screening mammogram. In a study by Taplin et al., self-reported benign breast biopsy was associated with statistically significantly reduced mammography performance, particularly in specificity, positive predictive value, and overall accuracy [18]. However, it is essential to note that intrinsic breast tissue characteristics, such as fibrocystic changes or fibroglandular breast structure, might also be associated with higher chances of requiring, and thus, undergoing a biopsy. 

Obesity and breast implants significantly decreased the odds of obtaining inconclusive screening mammograms. Prior studies have found that women with fatty and homogeneous breast tissue exhibit significantly higher screening sensitivity [17,19,20]. Other studies reported consistent results that compared to nonobese women, obese women are more likely to have fatty breast tissue [21,22]. Thus, mammograms of women with fatty breast composition might be less challenging to interpret than those with dense breast tissue. Breasts with higher fat content tend to have lower density, reducing the probability of receiving a BI-RADS 0 result. These findings align with the observation of higher BI-RADS 0 rates among younger women, whose breasts tend to have a lower proportion of fat tissue.

Breast augmentation decreased the odds of obtaining inconclusive screening mammography. To maximize the visualization of breast tissue, women with breast implants typically undergo two additional images per breast, known as implant displacement views, which might improve diagnostic accuracy [23]. A study encompassing seven mammography registries in the United States discovered that breast augmentation decreases the sensitivity of screening mammography [24]. However, the study did not provide information regarding whether there was a difference in the number of BI-RADS 0 results between the two groups. This variation in results may be attributed to the unique population on the LFMMC in Miami-Dade County and variations in patient demographics and breast implant characteristics specific to this geographic area. Further research on the role of breast augmentation on screening mammography accuracy is warranted to assess in further detail how breast implant imaging positioning, ruptures, leaks, interference, and artifacts influence the interpretation of the images.

The observed reduction in the odds of BI-RADS 0 with breast augmentation in our study prompts us to consider whether the inclusion of additional prior mammogram images for comparison could potentially mitigate the occurrence of inconclusive screening mammography results. Given that the LFMMC exclusively serves uninsured women, the increased rate of inconclusive mammograms could be attributed to the absence of reference images, as for many of the women, it might be their initial and sole mammogram. Due to the limited number of reference images, radiologists may encounter challenges in interpreting the presenting mammogram, potentially resulting in the classification of the mammogram as BI-RADS 0. It would be intriguing to analyze this specific data in future studies and investigate the possible association between the lack of reference images and inconclusive mammograms.

Our study findings emphasize several important implications for improving the accuracy and efficiency of breast cancer screening practices. Healthcare providers should integrate breast ultrasounds as a supplemental screening tool, particularly for young women with dense breast tissue [25]. Recent nationwide initiatives have emphasized the documentation of breast density for all women and suggest breast ultrasound in cases where the breast density is categorized as C or D. Notably, the state of Florida was an early adopter of this legislation, and our observations reveal a subsequent increase in the BI-RADS 0 rate. Furthermore, molecular imaging, such as positron emission mammography (PEM) [26,27] and diffusion-weighted imaging (DWI) [28], could also serve as a valuable adjunct for breast cancer screening in young women with dense breasts. Continued research into supplementing these modalities is needed before their implementation into clinical practice.

However, it's important to acknowledge that the current financial constraints, particularly in our mobile mammography center primarily serving uninsured patients, may make these expensive screening methods challenging. The LFMMC serves a population predominantly composed of racial minorities and low-income women, who experience disproportionately high breast cancer mortality rates often attributed to delayed screening. These women frequently encounter barriers, including lack of healthcare access, biological and genetic differences in tumors, variations in the prevalence of risk factors, and lack of insurance. Nonetheless, with increased funding or donations in the future, it may become feasible to incorporate these advanced screening methods into the mobile center's services, potentially enhancing breast cancer screening for underserved populations. Moreover, it is imperative to address the immediate need for access to follow-up diagnostic mammograms for patients with inconclusive findings, as failure to do so could compromise the timely detection and treatment of suspicious lesions. By assisting with scheduling diagnostic mammograms and ensuring prompt follow-up care for patients with inconclusive results, LFMMC mitigates the risks associated with delayed diagnosis and improves overall screening outcomes for the community. Leveraging the expertise of navigators has been particularly beneficial in facilitating access to follow-up care and navigating the healthcare system effectively.

Additionally, considering the implications of using artificial intelligence in screening mammography interpretations could further optimize the screening process. Incorporation of these additional imaging modalities alongside mammography could be assessed in future studies aimed at improving the effectiveness of breast cancer screening, particularly for this specific group of women on the LFMMC. Emphasis should also be placed on obtaining prior imaging whenever possible.

Limitations

Limitations of the study include unmeasured variables such as alcohol consumption, number of prior pregnancies, age of menarche, current menstrual cycle phase, and history of regular or irregular periods. Inadequately measured variables may have arisen due to the inclusion of additional questions in the intake forms over the years. Consequently, only 1,914 out of 5,000 patients were included in the formal analysis to ensure a comprehensive assessment. Another limitation is that the LFMMC only performs screening mammograms, which prevents radiologists from classifying mammograms with suspicious findings under an abnormal BI-RADS score (4 to 6). Thus, BI-RADS 0 results in screening mammography encompass two interpretations: 1) mammogram findings were unclear/inconclusive for other than suspicious findings (e.g., methodological difficulties), or 2) there was suspicion of malignant imaging. The high prevalence of BI-RADS 0 reported may stem from the complexity of images in the LFMMC, possibly secondary to a younger target population. Alternatively, and of more significant concern, there may have been a higher suspicion of breast malignancy in our population.

Further studies with detailed insights into the radiologist's rationale for assigning BI-RADS 0 in each patient's screening mammogram are essential for a more precise understanding of our population's elevated BI-RADS 0 rates. It is also important to note that the radiologist at LFMMC has remained consistent over the years, with only one radiologist conducting the interpretations. Additionally, there doesn't appear to be a correlation between the prevalence of BI-RADS 0 findings and the center's workload. Our technologists' proper training and adherence to protocols also ensure our mammogram results' accuracy and reliability. Technical recalls at LFMMC are minimal, and double reads for select cases have consistently yielded 100% congruence, as mandated by accreditation requirements. These factors indicate a high level of reliability and consistency in mammogram interpretations at LFMMC, suggesting that factors other than radiologist variability or workload may contribute to the high prevalence of BI-RADS 0 findings.

While associations of BI-RADS 0 findings with demographic and clinical characteristics provide valuable insights into potential risk factors, it is important to acknowledge that they may not directly address the mobile center's issue of the high prevalence of BI-RADS 0 findings. Instead, these associations offer valuable context and understanding of the factors contributing to inconclusive mammogram results. To effectively address the high BI-RADS 0 prevalence, additional strategies such as implementing follow-up protocols for patients with inconclusive findings, providing access to diagnostic mammograms, and potentially incorporating complementary imaging modalities may be necessary. These interventions can help mitigate the challenges posed by inconclusive results and improve the overall effectiveness of breast cancer screening at the mobile center. Ongoing research and proactive measures can lead to more equitable and accessible breast cancer screening, ultimately enhancing women's health outcomes.

## Conclusions

Our study sheds light on the higher frequency of BI-RADS 0 in our local mammography center compared to the national average. Younger age, premenopausal status, single status, and history of a benign biopsy increase the odds of inconclusive mammograms, while obesity and breast implants decrease those odds. This highlights the unique challenges faced by disproportionately affected populations, including racial minorities and low-income women, in accessing timely and conclusive breast cancer screening. While implementing advanced screening methods may be hindered by financial constraints, addressing the immediate need for follow-up diagnostic mammograms and leveraging the expertise of navigators are crucial steps in improving screening outcomes. Additionally, exploring the potential of artificial intelligence and emphasizing the importance of prior imaging could optimize the screening process, ultimately advancing women's health outcomes in underserved communities.
